# Relationship between intima-media thickness of the common carotid artery and arterial stiffness in subjects with and without type 2 diabetes: a case-series report

**DOI:** 10.1186/1475-2840-10-3

**Published:** 2011-01-12

**Authors:** Manuel Ángel Gómez-Marcos, José Ignacio Recio-Rodríguez, María Carmen Patino-Alonso, Cristina Agudo-Conde, Leticia Gómez-Sánchez, Emiliano Rodríguez-Sánchez, Carlos Martín-Cantera, Luís García-Ortiz

**Affiliations:** 1 Primary Care Research Unit, La Alamedilla Health Center, Salamanca, Spain; 2 Statistics Department, University of Salamanca, Salamanca, Spain; 3 Department of Medicine, Barcelona Autonomous University, Barcelona, Spain

## Abstract

**Background:**

We examined the relationship between the intima-media thickness of the common carotid artery (CCA-IMT) and arterial stiffness, assessed by pulse wave velocity (PWV), the ambulatory arterial stiffness index (AASI) and the augmentation index (AIx) in subjects with and without type 2 diabetes.

**Methods:**

A case-series study was made in 366 patients (105 diabetics and 261-non-diabetics). Ambulatory blood pressure monitoring was performed on a day of standard activity with the SpaceLabs 90207 system. AASI was calculated as "1-slope" from the within-person regression of diastolic-on-systolic ambulatory blood pressure readings. PWV and AIx were measured with the SphygmoCor system, and a Sonosite Micromax ultrasound unit was used for automatic measurements of CCA-IMT.

**Results:**

PWV, AASI and CCA-IMT were found to be greater in diabetic patients, while no differences in AIx were observed between the two groups. CCA-IMT was independently correlated to the three measures of arterial stiffness in both groups. We found an increase in CCA-IMT of 0.40, 0.24 and 0.36 mm in diabetics, and of 0.48, 0.17 and 0.55 mm in non-diabetics for each unit increase in AASI, AIx and PWV. The variability of CCA-IMT was explained mainly by AASI, AIx and gender in diabetic patients, and by age, gender, AASI and PWV in non-diabetic patients.

**Conclusions:**

CCA-IMT showed a positive correlation to PWV, AASI and AIx in subjects with and without type 2 diabetes. However, when adjusting for age, gender and heart rate, the association to PWV was lost in diabetic patients, in the same way as the association to Alx in non-diabetic patients. The present study demonstrates that the three measures taken to assess arterial stiffness in clinical practice are not interchangeable, nor do they behave equally in all subjects.

## Introduction

The ultrasound measurement of the intima-media thickness of the common carotid artery (CCA-IMT) has been recognized as a powerful method for identifying subclinical atherosclerosis. CCA-IMT is well-known to be a strong predictor of future vascular events and a surrogate marker of atherosclerosis [1-3].

Arterial stiffness assessed through pulse wave velocity (PWV) [4,5] and the ambulatory arterial stiffness index (AASI) [6,7]) is regarded as an independent predictor of cardiovascular mortality and morbidity in patients with cardiovascular disease as well as in healthy individuals. However, the data on the central Augmentation index (AIx) are contradictory [8] and its role in the clinical setting remains unclear. Several studies suggest that the methods would not be interchangeable in a clinical setting [9-12].

Intima-media thickness (IMT) is associated with PWV and AASI in untreated hypertensive patients [13]. The studies made in type 2 diabetic patients conclude that PWV is associated to microalbuminuria [14] and renal function estimated by the glomerular filtration rate [15]. However, there are no data on the relationship between IMT and arterial stiffness at central level. A recent study in a Japanese population with type 2 diabetes found CCA-IMT, but not PWV, to be independently associated to silent cerebral infarction [3].

The present study examines the relationship between CCA-IMT and arterial stiffness evaluated by PWV, AASI and AIx in subjects with and without type 2 diabetes.

## Materials and methods

Study design and population: A case-series study was conducted in a primary care setting. Using consecutive sampling, we included 366 patients (105 diabetics and 261 non-diabetics) from a population of 46,000 people corresponding to two primary care centers. The patients were between 30-80 years old, visited their family doctor between January 2.009 and June 2.010, and signed the informed consent to participation in the study. The latter adhered to the principles of the Declaration of Helsinki, and was approved by an independent ethics committee of Salamanca University Hospital (Spain).

### Measurement

A detailed description of the measurement techniques can be found elsewhere [16]. Clinical blood pressure was determined by performing three systolic blood pressure (SBP) and diastolic blood pressure (DBP) measurements with a validated sphygmomanometer (OMRON M7, Omron Health Care, Kyoto, Japan), following the recommendations of the European Society of Hypertension [17]. The mean of the last two measurements obtained by the nurse of the research unit was used for the study.

Ambulatory blood pressure monitoring (ABPM) was performed on a day of standard activity, using a cuff suited to the size of the patient's arm. A SpaceLabs 90207 control system (Spacelabs Healthcare, Issaquah, Washington, USA), validated according to the protocol of the British Hypertension Society, was used [18]. The records of readings considered to be valid were ≥ 66% of the total. Furthermore, in order for the records to be evaluable, at least 14 measurements were required during the daytime period, or at least 7 during the nighttime or rest period. The monitor was programmed to obtain blood pressure recordings every 20 min. during the daytime period and every 30 min. during the rest period.

### Ambulatory arterial stiffness index

AASI was calculated as 1 minus the regression slope of diastolic blood pressure (DBP) plotted against systolic blood pressure (SBP) obtained through individual 24-hour blood pressure monitoring [19]. The stiffer the arterial tree, the closer the regression slope and AASI are to 0 and 1, respectively [20,21].

### Pulse wave analysis (PWA) and pulse wave velocity (PWV)

AIx and PWV were estimated using the SphymgoCor System (AtCor Medical Pty Ltd Head Office, West Ryde, Australia), currently accepted as the gold standard for measuring arterial stiffness. Using the SphygmoCor System (Px Pulse Wave Analysis), with the patient in the sitting position and resting the arm on a rigid surface, pulse wave analysis was performed with a sensor in the radial artery, using mathematical transformation to estimate the aortic pulse wave. From the morphology of the aortic wave, central (aortic) blood pressure, the pressure increase, and central pressure pulse were estimated. Central AIx was estimated using the following formula: increase in central pressure * 100/pulse pressure. Using the SphygmoCor System (Vx Pulse Wave Velocity), and with the patient in the supine position, the pulse wave of the carotid and femoral arteries was analyzed, estimating the delay with respect to the ECG wave and calculating the PWV. Distance measurements were taken with a measuring tape from the sternal notch to the carotid and femoral arteries at the sensor location [16]. A measurement of greater than 12 m/s was considered to indicate subclinical organ damage (SOD) [22].

### Assessment of carotid intima-media thickness (IMT)

Carotid ultrasound for assessing IMT was performed by two investigators specifically trained to the effect before starting the study. The reliability of the measurements was evaluated before the start of the study using the intraclass correlation coefficient, which showed values of 0.97 for intraobserver agreement on repeated measurements in 20 subjects, and 0.89 for interobserver agreement. A Sonosite Micromax ultrasound device coupled to a 5-10 MHz multifrequency high-resolution linear transducer with Sonocal software was used for performing automatic measurements of IMT in order to optimize reproducibility. Measurements were made of the common carotid after the examination of a 10-mm longitudinal section at a distance of 1 cm from the bifurcation, performing measurements in the anterior or proximal wall, and in the posterior or distal wall in the lateral, anterior and posterior projections, following an axis perpendicular to the artery to discriminate two lines: one for the intima-blood interface and the other to the media-adventitious interface. A total of 6 measurements were obtained of the right carotid and another 6 of the left carotid, using average values (average IMT) and maximum values (maximum IMT) calculated automatically by the software. The measurements were obtained with the subject lying down, with the head extended and slightly turned opposite to the examined carotid, following the recommendations of the Manheim Carotid Intima-Media Thickness Consensus [23]. Mean IMT was considered to be abnormal if above 0.9 mm, or if there were atherosclerotic plaques with a diameter of over 1.5 mm, or a focal increase of 0.5 mm, or 50% of the adjacent IMT [22].

### Statistical analysis

Continuous variables were expressed as the mean ± standard deviation, while frequency distributions were used in application to qualitative variables. The mean difference between two categories of qualitative variables was analyzed with the Student's t-test for independent samples. Pearson's correlation coefficient was used to estimate the relationship between the quantitative variables, while the chi-square test was used to associate the qualitative variables. We performed multiple linear regression analysis by means of the enter method, using CCA-IMT as dependent variable -and parameters such as age, gender (male = 1; female = 0), office heart rate, AASI, AIx and PWV as independent variables. In order to obtain a homogeneous measurement scale and facilitate interpretation of the regression analysis, standardized variables were used. The data were analyzed using the SPSS version 17.0 statistical package (SPSS Inc., Chicago, Illinois, USA). A value of P < 0.05 was considered statistically significant.

## Results

The main clinical characteristics of the patients (105 diabetics and 261 non-diabetics) are reported in Table 1. AASI, PWV, CCA-IMT and the percentage of patients presenting subclinical organ damage (SOD) in carotid arteries were higher in diabetic patients, though no differences in AIx were observed between the two groups.

**Table 1:** General demographic and clinics characteristics: in subjects with and without type 2 diabetes

Age*	55.07 ± 11.92	59.81 ± 10.20	53.32 ± 12.05
Male, n (%) Female, n (%)	220 (61.08) 136 (38.20)	65 (67.70) 31 (32.30)	155 (59.60) 105 (40.40)
Smokers, n (%)	84 (23.60)	20 (20.80)	64 (24.60)
Ischemic heart disease, n (%)*	25 (7.00)	15 (15.60)	10 (3.80)
Cerebrovascular disease, n (%)	6 (1.70)	2 (2.10)	4 (1.50)
Body mass index, kg/m2*	28.48 ± 4.35	29.97 ± 5.24	27.93 ± 3.84
Waist circumference, (cm)*	97.52 ± 11.97	102.58 ± 12.51	95.66 ± 11.23
Total Cholesterol, (mg/dl)*	203.55 ± 37.05	188.00 ± 33.26	209.33 ± 36.77
Tryglicerides, (mg/dl)	131.10 ± 77.10	143.11 ± 86.09	126.63 ± 73.16
LDL cholesterol, (mg/dl)*	125.24 ± 32.63	109.94 ± 26.53	130.92 ± 32.89
HDL cholesterol,(mg/dl)*	51.96 ± 12.58	48.32 ± 10.75	53.32 ± 12.96
Serum glucose, (mg/dl)*	99.54 ± 30.00	132.00 ± 39.79	87.51 ± 10.71
Serum creatinine, (mg/dl)	0.88 ± 0.18	0.85 ± 0.15	0.89 ± 0.18
Office SBP, mm Hg	139.26 ± 17.24	137.56 ± 18.43	139.89 ± 16.77
Office DBP, mm Hg*	86.88 ± 10.03	83.03 ± 11.22	88.31 ± 10.64
Office PP, mm Hg	52.95 ± 13.10	55.11 ± 13.63	52.16 ± 12.84
HR office bpm	72.26 ± 12.75	72.10 ± 12.97	72.37 ± 12.69
24-hour SBP, mm Hg*	125.79 ± 12.80	123.43 ± 12.60	126.66 ± 12.79
24-hour DBP, mm Hg*	77.01 ± 9.88	72.60 ± 8.66	78.64 ± 9.82
24-hour PP, mm Hg*	48.78 ± 9.68	50.83 ± 10.06	48.02 ± 9.45
Night/day ratio SBP	0.88 ± 0.07	0.90 ± 0.07	0.88 ± 0.08
Night/day ratio DBP*	0.83 ± 0.09	0.85 ± 0.09	0.83 ± 0.09
AASI*	0.38 ± 0.06	0.40 ± 0.06	0.37 ± 0.05
PWV, (m/s)*	9.00 ± 2.23	9.91 ± 2.34	8.68 ± 2.10
AIx	30.27 ± 11.49	30.77 ± 11.14	30.09 ± 11.63
Carotid IMT, mm*	0.73 ± 0.11	0.77 ± 0.11	0.71 ± 0.11
Carotid SOD, n (%)*	53 (14.90)	25 (26.00)	28 (10.80)

Data for qualitative variables are expressed as n (%) and quantitative variables as mean ± standard deviation. SBP: systolic blood pressure; DBP: dyastolic blood pressure; PP: pulse pressure; HR: heart rate; bpm: beats per minute; LDL: low density lipoprotein; HDL: high density lipoprotein; AASI: ambulatory arterial stiffness index; AIx: augmentation index; PWV: pulse wave velocity; IMT: intima-media thickness; SOD: subclinical organ damage. p:statistically differences between subjects with type 2 diabetes and non-diabetic. *p < 0.05.

The existing correlations of PWV, AASI and AIx to patient age, cardiovascular risk factors and the different measures of clinical blood pressure and blood pressure are shown in Table 2. An increase in age, 24-hour blood pressure monitoring and CCA-IMT were associated with a rise in the three measurements of arterial stiffness in both groups. An increase in the night/day ratio of SBP and DBP was associated to an increase in AASI and PWV in the two groups; however, there was no correlation to AIx in either of them. Office and ambulatory blood pressure showed a positive correlation to PWV and AASI, though only ambulatory pressure monitoring was correlated to AIx. The latter was not associated to PWV in diabetic patients, or to AASI in non-diabetic patients. However, carotid CCA-IMT showed a positive correlation to all the arterial stiffness parameters.

**Table 2:** Univariate Pearson correlations between IMT, risk factors and arterial stiffness measures (ambulatory arterial stiffness index, augmentation index and pulse wave velocity) in subjects with and without type 2 diabetes

	Ambulatory arterial stiffness index	Augmentation index	Pulse wave velocity	Carotid IMT, mm
**Univariate**	**Correlation coefficient**	**Correlation coefficient**	**Correlation coefficient**	**Correlation coefficient**

		**Diabetics**		

Age	0.45**	0.30**	0.48**	0.41**
Body mass index, kg/m^2^	0.08	-0.08	0.20	0.04
Waist circumference, (cm)	0.13	-0.13	0.30**	0.21*
Total Cholesterol, (mg/dl)	-0.09	-0.02	0.09	0.04
Tryglicerides, (mg/dl)	-0.14	-0.03	0.11	-0.02
LDL cholesterol, (mg/dl)	-0.14	-0.05	-0.02	0.09
HDL cholesterol, (mg/dl)	0.17	0.16	0.08	-0.13
Office PP, mm Hg	0.65**	0.03	0.52**	0.36**
HR office bpm	-0.24	-0.44**	0.09	-0.14
24-hour PP, mm Hg	0.87**	0.25*	0.44**	0.37**
Night/day ratio SBP	0.33**	0.11	0.19	0.03
Night/day ratio DBP	0.39**	0.05	0.22*	0.05
Carotid IMT, mm	0.35**	0.22*	0.36**	1
AASI	1	0.24*	0.41**	0.35**
AIx	0.24*	1	0.08	0.22*
PWV m/s	0.41**	0.08	1	0.36**

	**No Diabetics**			

Age	0.43**	0.38**	056**	0.67**
Body mass index, kg/m^2^	0.08	-0.17**	0.17**	0.12
Waist circumference, (cm)	0.01	-0.21**	0.22**	0.20**
Total Cholesterol, (mg/dl)	-0.04	0.09	0.08	0.10
Tryglicerides, (mg/dl)	-0.10	-0.08	0.06	0.04
LDL cholesterol, (mg/dl)	-0.04	0.05	0.10	0.10
HDL cholesterol, (mg/dl)	0.08	0.22**	-0.07	-0.06
Office PP, mm Hg	0.58**	0.11	0.32**	0.38**
HR office bpm	-0.19**	-0.25**	0.05	-0.17**
24-hour PP, mm Hg	0.85**	0.14*	0.34**	0.43**
Night/day ratio SBP	0.27**	0.13*	0.19**	0.30**
Night/day ratio DBP	0.27**	0.13*	0.17**	0.23**
Carotid IMT, mm	0.42**	0.17**	0.49**	1
AASI	1	0.09	0.30**	0.42**
AIx	0.09	1	0.23**	0.17**
PWV m/s	0.30**	0.23**	1	0.50**

LDL: low density lipoprotein; HDL: high density lipoprotein; MBP: mean blood pressure; PP: pulse pressure; SBP: syistolic blood pressure; DBP: dyastolic blood pressure; HR: heart rate; bpm: beats per minute; IMT: intima-media thickness. AASI: ambulatory arterial stiffness index; AIx: augmentation index; PWV: pulse wave velocity. *p: p < 0.05.**p < 0.01.

**Figure 1:**
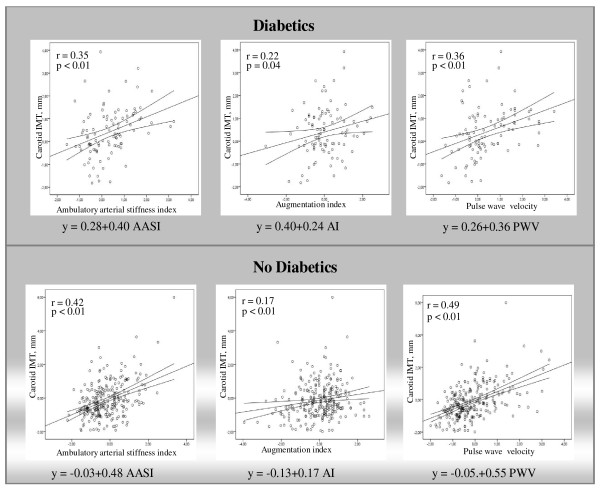
**Simple linear regression between intima-media thickness of the common carotid artery and arterial stiffness parameters: pulse wave velocity (PWV), the ambulatory arterial stiffness index (AASI) and the augmentation index (AIx), in subjects with and without type 2 diabetes**. Parallel lines reflect the regression line of the relationship with 95% confidence intervals.

Figure 1 shows the simple linear regression straight line of CCA-IMT with the parameters that assess arterial stiffness. In diabetic patients, CCA-IMT increased by 0.40, 0.24 and 0.36 mm per each unit increase in AASI, AIx and PWV. In non-diabetic patients, CCA-IMT increased by 0.48, 0.17 and 0.55 mm per each unit increase in AASI, AIx and PWV. However, diabetic patients were derived from more elevated values of CCA-IMT. The variability of CCA-IMT in diabetic patients was mainly explained by AASI, AIx and gender; age, PWV and heart rate were not significant. However, in non-diabetic subjects CCA-IMT variability was explained by age, gender, AASI and PWV, and only AIx and heart rate had no significant influence (Table 3).

**Table 3:** Multiple Linear Regression Analysis: Relationship Between Carotid IMT and Selected Variables: in subjects with and without type 2 diabetes

Variable	Not standardized β	Confidence interval 95%	*P*
**Diabetics**			

Dependent variable: Carotid IMT (Adjusted R^2 ^= 0.266)			

Constante	-0.43	-0.86 a -0.01	0.05
Age	0.28	-0.01 a 0.56	0.06
Gender	0.80	0.28 a 1.33	p < 0.01
HR office mpb	0.16	-0.12 a 0.43	0.26
PWV	0.11	-0.12 a 0.33	0.36
AASI	0.31	0.05 a 0.57	0.02
Augmentation index	0.29	0.02 a 0.55	0.03

**No Diabetics**			

Dependent variable: Carotid IMT (Adjusted R^2 ^= 0.520)			

Constante	-0.34	-0.50 a -0.18	p < 0.01
Age	0.55	0.43 a 0.68	p < 0.01
Gender	0.46	0.25 a 0.68	p < 0.01
HR office mpb	-0.04	-0.14 a 0.06	0.44
PWV	0.17	0.05 a 0.29	p < 0.01
AASI	0.20	0.08 a 0.31	p < 0.01
Augmentation index	-0.01	-0.19a 0.11	0.91

Dependent variable: Carotic IMT: Carotic Intima-media thickness. Independent variables: Age; Gender: (male = 1; female = 0); HR: Heart rate; mbp: beats per minute AASI: Ambulatory arterial stiffness index; PWV: pulse wave velocity. R2: determination coefficient; p: statistically significant differences (p < 0.05).

## Discussion

The present study provides the first systematic evaluation of the relationship between CCA-IMT and arterial stiffness in subjects with type 2 diabetes and in non-diabetics. We found that the values of AASI, PWV and CCA-IMT, but not AIx, are higher in diabetic subjects. There is a positive correlation between CCA-IMT and the parameters used to assess arterial stiffness. However, AASI would explain the variability of CCA-IMT in both groups, AIx only in diabetic patients, and PWV in non-diabetic subjects.

In the same way as in patients with metabolic syndrome, where according to the CARMELA study [24] CCA-IMT increases with the number of existing risk factors, patients with type 2 diabetes show higher CCA-IMT values.

As in other studies which compare subjects with and without type 2 diabetes [25], patients with or without renal disease [9] and healthy people versus hypertensive patients [11], we found no differences in AIx between subjects with and without type 2 diabetes. These results support the conclusion of Lacy et al. [25] who found PWV to be increased in people with diabetes, but this was not associated to increased AIx. These findings conclusively demonstrate that AIx is not a reliable measure of arterial stiffness in people with diabetes. This is very likely to also apply to other population groups, such as individuals with hypertension and renal disease.

In our univariate analysis AIx was not associated to PWV (r = 0.08, p = 0.46) in diabetic patients, or to AASI (r = 0.09, p = 0.14) in non-diabetic patients. Li et al. [19] reported a correlation between AIx and AASI of r = 0.48 and between PWV and AASI of r = 0.51. These values are higher than those found in our study, but it must be remembered that the study populations are different, and the behavior of the parameters used to assess arterial stiffness might not be the same. The three parameters present a positive correlation to age in both groups, a fact already described in other studies [11,26].

The association between AASI and increased CCA-IMT is an ongoing finding in hypertensive patients [13]; in this context, the coefficient reported by Leoncini et al. [27] was (r = 0.20), which is lower than the value described by Garcia et al. [28] (r = 0.42), and higher than the coefficient found in our study both in diabetics (r = 0.31) and in non-diabetics (r = 0.20). PWV in turn is associated to CCA-IMT in hypertensive patients [13], whereas in diabetic patients Ito et al. [29] found a correlation (r = 0.22) higher than that obtained in our study (r = 0.15) in this group of patients.

The correlation between AIx and CCA-IMT reported by Westerbacka et al. [30] in diabetics (r = 0.16; p < 0.01) was lower than that recorded in our study in type 2 diabetics (r = 0.20) and in non-diabetics (r = 0.17). However, in the multiple regression analysis, on adjusting for age, gender and heart rate, the AIx beta value lost statistical significance in the group of patients without diabetes. This supports the idea that AIx as a measure of arterial stiffness does not behave in the same way in subjects with and without type 2 diabetes.

In a recently published study among untreated hypertensive patients, Triantafyllidi [13] assessed the relationship between the arterial stiffness values and PWV and AASI and the presence of target organ damage. On examining CCA-IMT, it was concluded that the simultaneous estimation of three noninvasive indexes of arterial stiffness (PWV, AASI and office pulse pressure) generates valuable information regarding their association with SOD in never-treated hypertensive patients in reference to their dipping status.

In a study carried out by Polak et al. [31] to analyze the influence of the different cardiovascular risk factors upon IMT, patient age and gender were found to account for 23.5% of the variability of CCA-IMT the next most important factor being systolic blood pressure.

Baumann et al. [32], in patients with normal blood glucose levels, found carboxymethyl lysine to be associated to an increased carotid artery diameter mainly in hypertensive patients, but no correlation to other arterial stiffness parameters was observed.

The main limitation of this study is its case series study, which hinders longitudinal analysis between CCA-IMT and AASI, PWV and AIx. Another restriction is the selection of the study population, based on consecutive sampling with pragmatic and broad inclusion criteria. Therefore, in the analyzed population we find diabetic and non-diabetic subjects with different treatment and evolution times. These facts may limit the external validity of the study. However, it must be remembered that the study sample represents the general population seen in primary care centers.

CCA-IMT showed a positive correlation to PWV, AASI and AIx in subjects with type 2 diabetes and in non-diabetics. However, on adjusting for gender, age and heart rate, the association with PWV and AIx was lost in diabetic patients and in non-diabetics, respectively. The present study demonstrated that in these patients there is poor agreement between AASI, PWV and AIx, confirming that the three measurements used to assess arterial stiffness in clinical practice are not interchangeable, nor do they behave equally in all subjects.

Lastly, we consider that follow-up studies are necessary to establish the relationship between CCA-IMT and each of these three measures, and to determine whether their joint determination offers additional benefits.

## Abbreviations

CCA-IMT: Intima-media thickness of the common carotid artery; PWV: Pulse wave velocity; AASI: Ambulatory arterial stiffness index; AIx: Augmentation index; SBP: Systolic blood pressure; DBP: Diastolic blood pressure; ABPM: Ambulatory blood pressure monitoring; PP: Pulse pressure; SOD: Subclinical organ damage;

## Competing interests

The authors declare that they have no competing interests.

## Authors' contributions

MAGM devised the study, designed the protocol, participated in fund raising, interpretation of results, prepared the manuscript draft and corrected the final version of the manuscript. JIRR and CAC participated in the study design, data collection and manuscript review. MCPA performed all analytical methods, interpretation of results, and manuscript review. ERS, LGS and CMC participated in the study design, interpretation of results, and manuscript review. LGO participated in the protocol design, fund raising, analysis of results, and final review of the manuscript. Finally, all authors reviewed and approved the final version of the manuscript.
